# Supplementary material for: Accurate ethnicity prediction from placental DNA methylation data
Source: Epigenetics Chromatin. 2019 Aug 9;12:51. doi: 10.1186/s13072-019-0296-3 (PMC6688210; doi:10.1186/s13072-019-0296-3)
Supplement: Supplementary file 2 — Additional file 2: Figure S1. Dataset-specific effects. PC1 by PC2 scatterplot from PCA computed on scaled and centered DNAme beta values from 499 samples and 319,233 sites. Figure S2. Performance between machine learning algorithms in training. Resampling results for each machine learning algorithm. a performance (LogLoss) between machine learning algorithms in predicting ethnicity, and b class-specific accuracy. Figure S3. Threshold analysis for determining “ambiguous” samples. Various cutoffs for predicted membership probabilities were compared with respect to changes in predictive performance. Figure S4. Dataset-specific performance. PlaNET’s classification performance was calculated for each dataset using a model trained to all other datasets. Figure S5. Enrichment analysis on ethnicity-predictive HM450K sites. PlaNET’s CpG sites used to predict ethnicity was tested for enrichment with respect to a chromosomal location, and b relation to CpG islands. Figure S6. Association of population structure PCs with technical and biological variables. PCs were computed on ethnicity-predictive sites, EPISTRUCTURE, Barfield’s method and the 59 SNP probes. Each PC was tested for their association with various cohort-specific technical and biological variables. For a given cohort (e.g. C1), ethnicity predictive sites from a classifier trained on all other cohorts (e.g. C2–C5) was used to avoid bias. Figure S7. PlaNET vs. Zhou et al. [10] snp-based classifier. PlaNET‘s ethnicity classification performance was compared to Zhou et al. [76] SNP-based ethnicity classifier in cohorts C3, C4, and C5. Figure S8. Estimating k number of ancestral populations using in genetic admixture inference program LEA. The cross-entropy criterion was used to determine the number of ancestral populations for estimating genetic ancestry coefficients. The number of ancestral populations was chosen at the point k = 3, when the cross-entropy criterion decreases significantly less with each integer-incr [file 13072_2019_296_MOESM2_ESM.pdf]

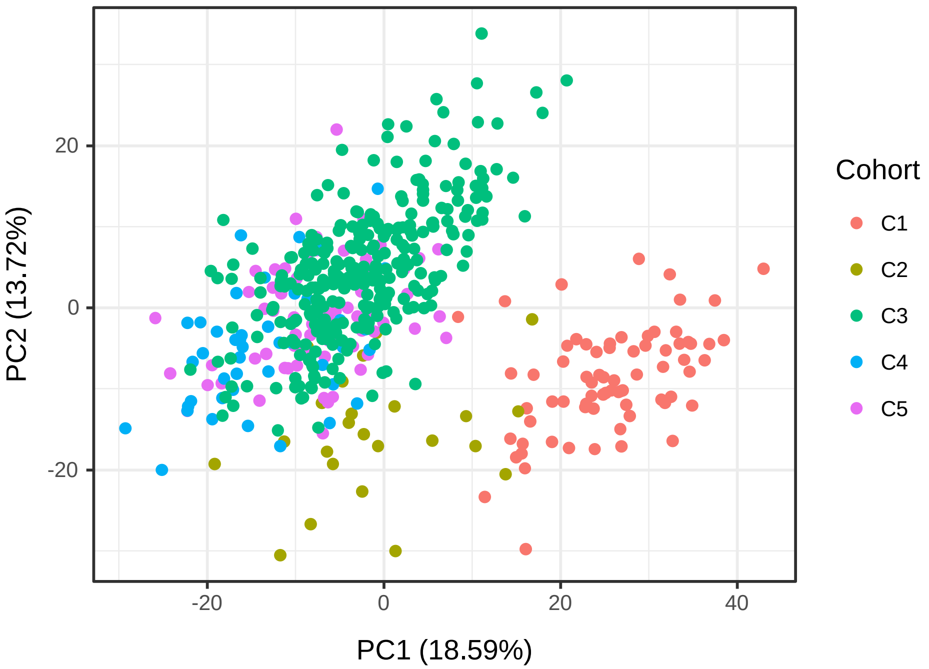

Figure S1. Dataset-specific effects. PC1 by PC2 scatterplot from PCA computed on scaled and centered DNAme beta values from 499 samples and 319233 sites.

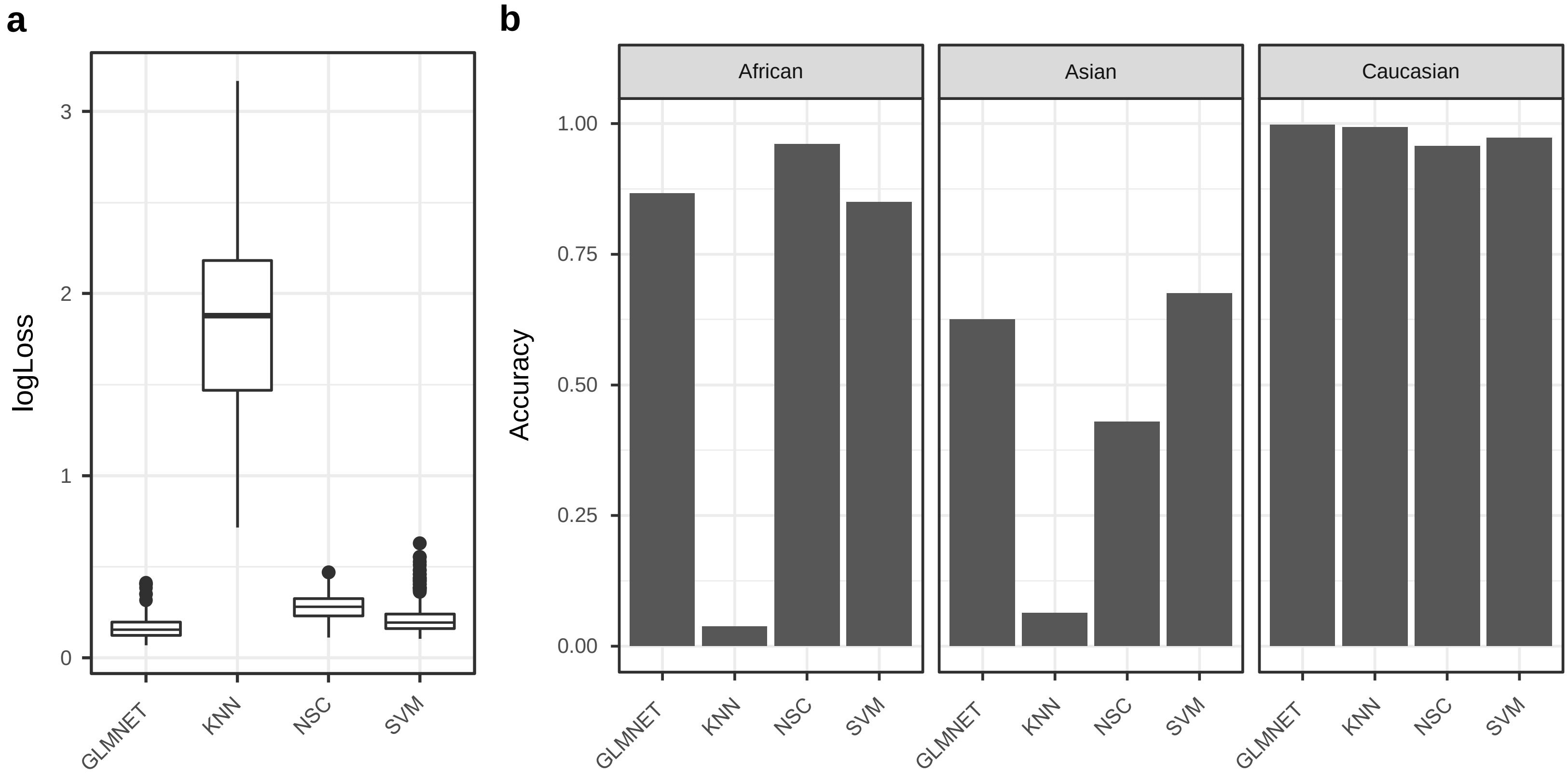

Figure S2. Performance between machine learning algorithms in training. Resampling results for each machine learning algorithm. **a** performance (LogLoss) between machine learning algorithms in predicting ethnicity, and **b** class-specific accuracy.

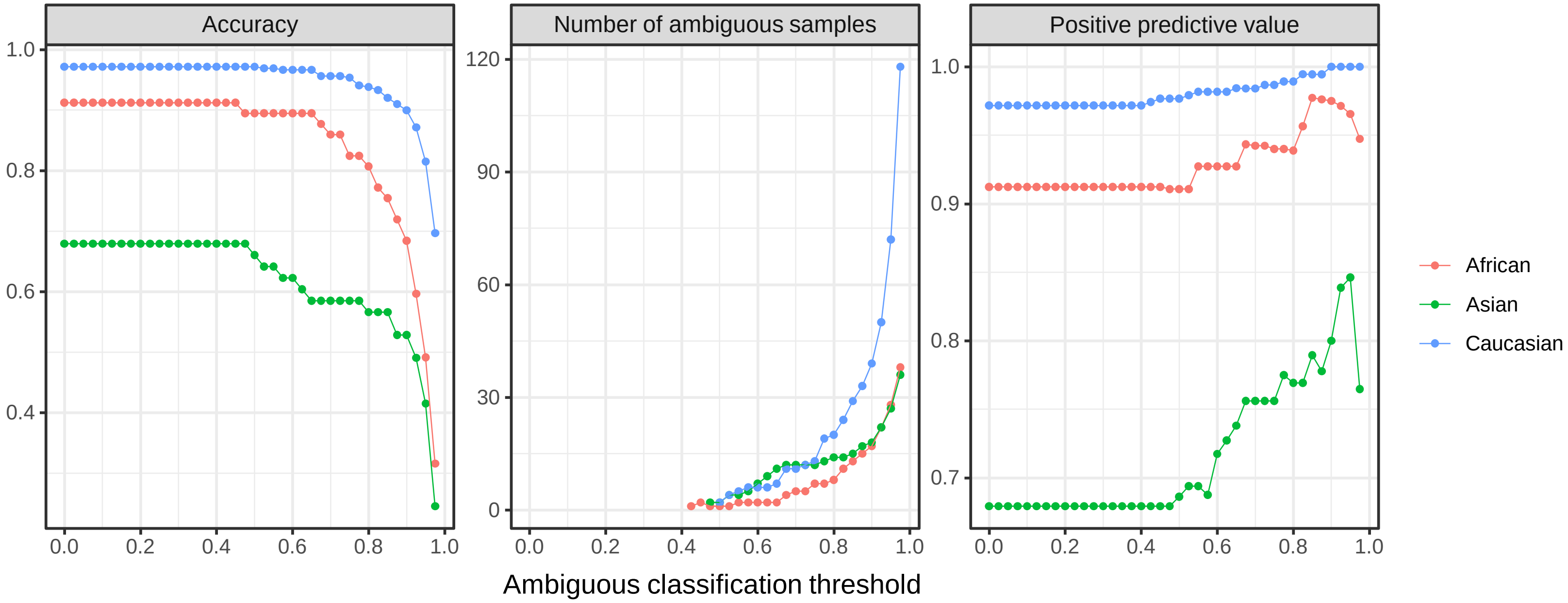

Figure S3. Threshold analysis for determining "ambiguous" samples. Various cutoffs for predicted membership probabilities were compared with respect to changes in predictive performance.

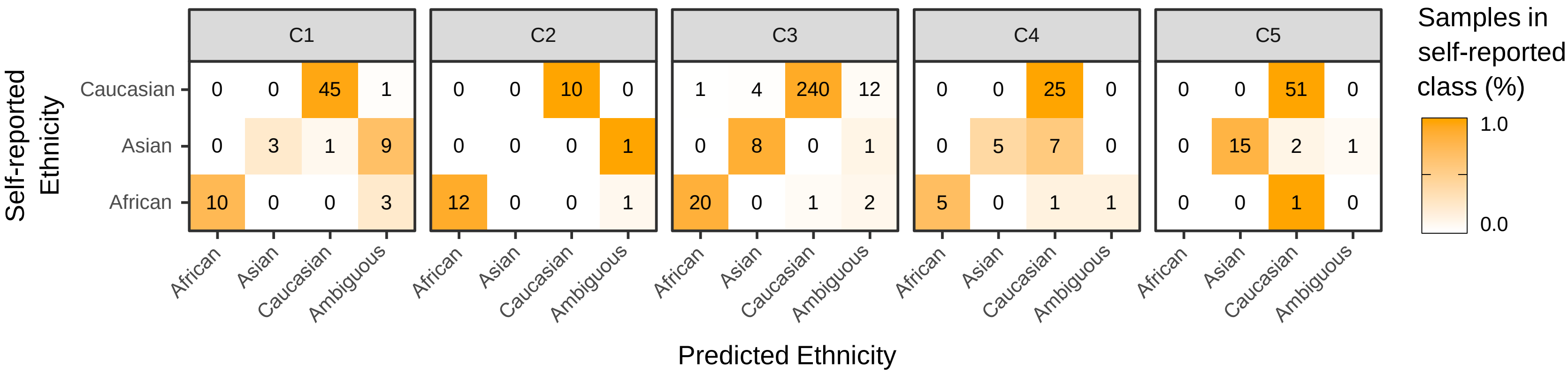

Figure S4. Dataset-specific performance. PlaNET's classification performance was calculated for each dataset using a model trained to all other datasets.

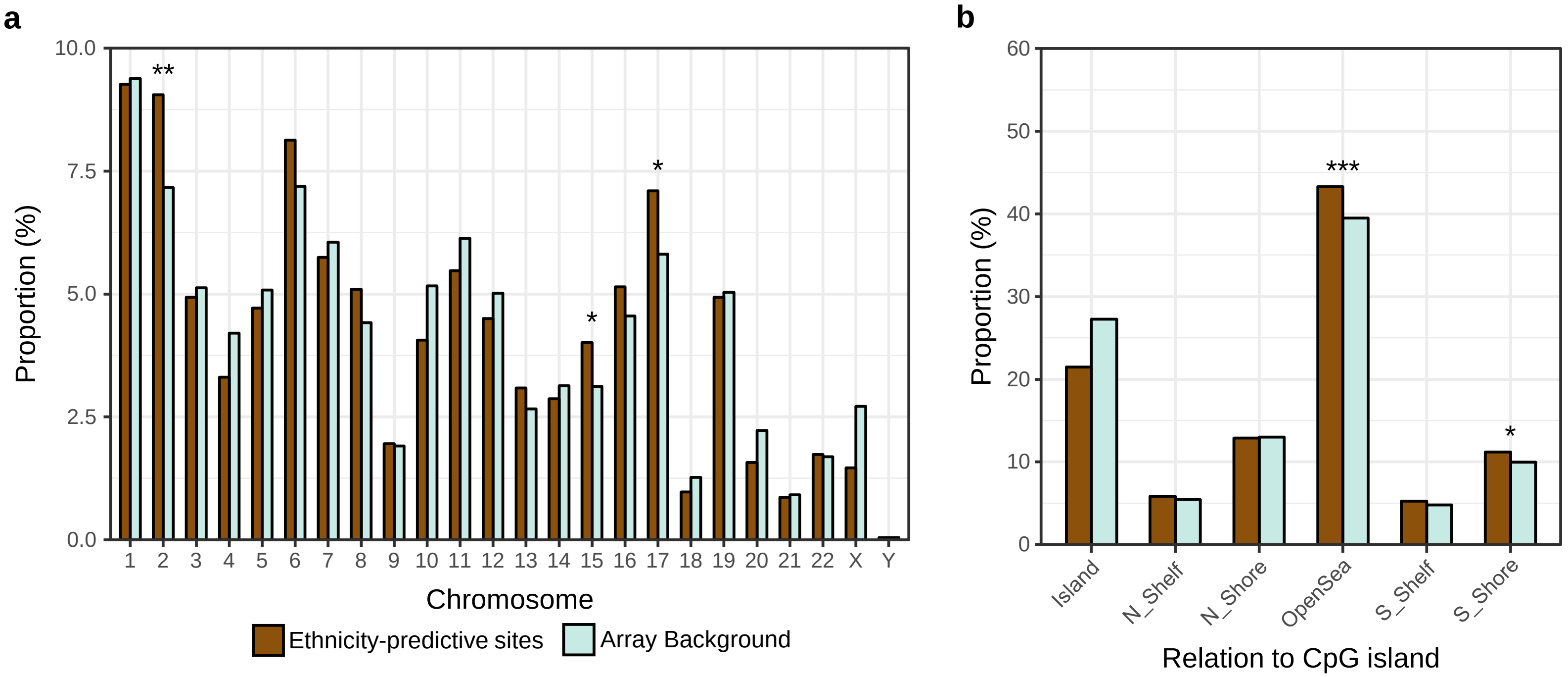

Figure S5. Enrichment analysis on ethnicity-predictive HM450K sites. PlaNET's CpG sites used to predict ethnicity was tested for enrichment with respect to **a** chromosomal location, and **b** relation to CpG islands.

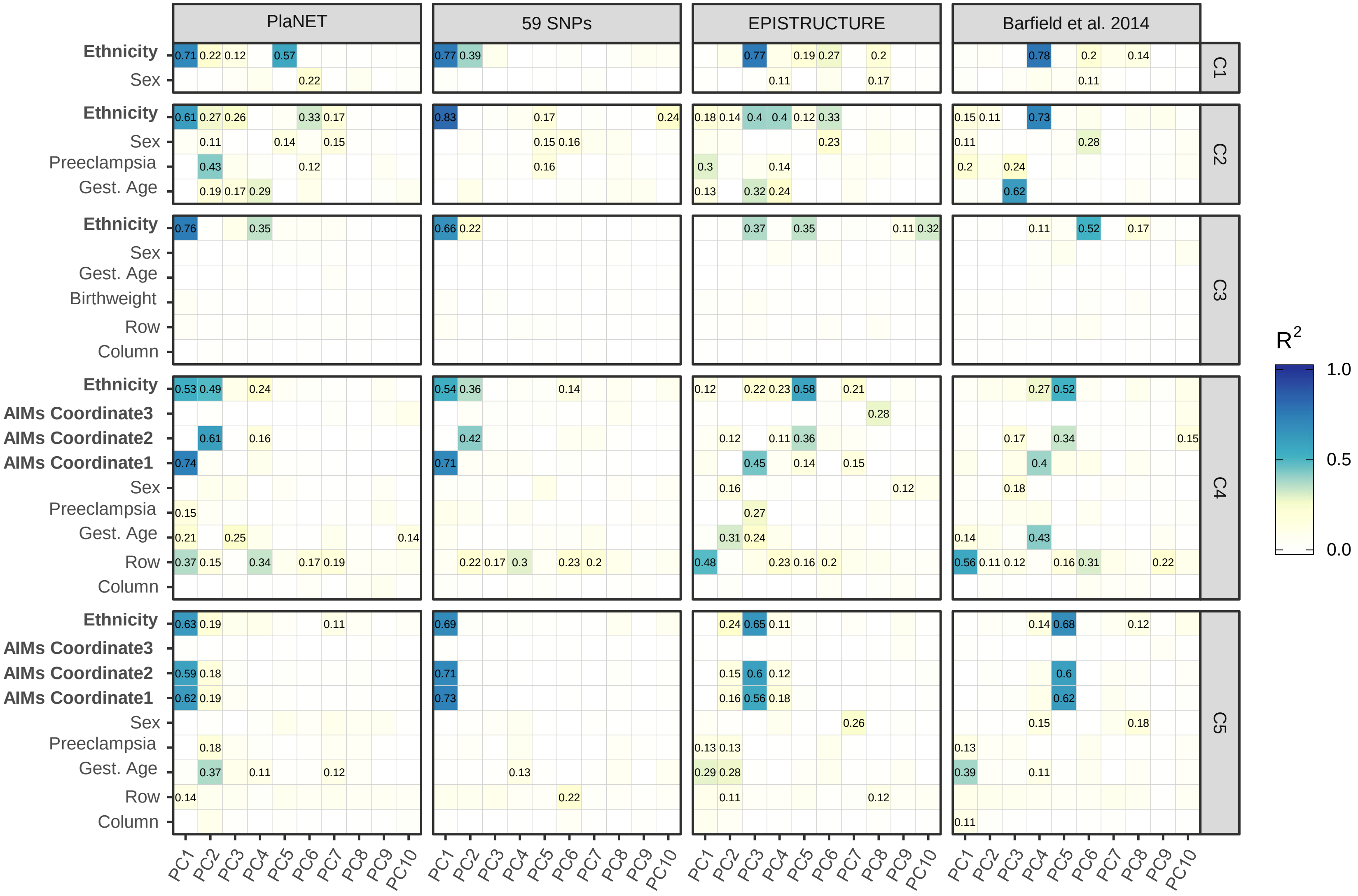

Figure S6. Association of population structure PCs with technical and biological variables. PCs were computed on the following sets of HM450K sites: ethnicity-predictive sites, 59 SNPs, EPISTRUCTURE, and Barfield's method. Each PC was tested for their association with various cohort-specific technical and biological variables. For a given cohort (e.g. C1), ethnicity predictive sites from a classifier trained on all other cohorts (e.g. C2-C5) was used to avoid bias.

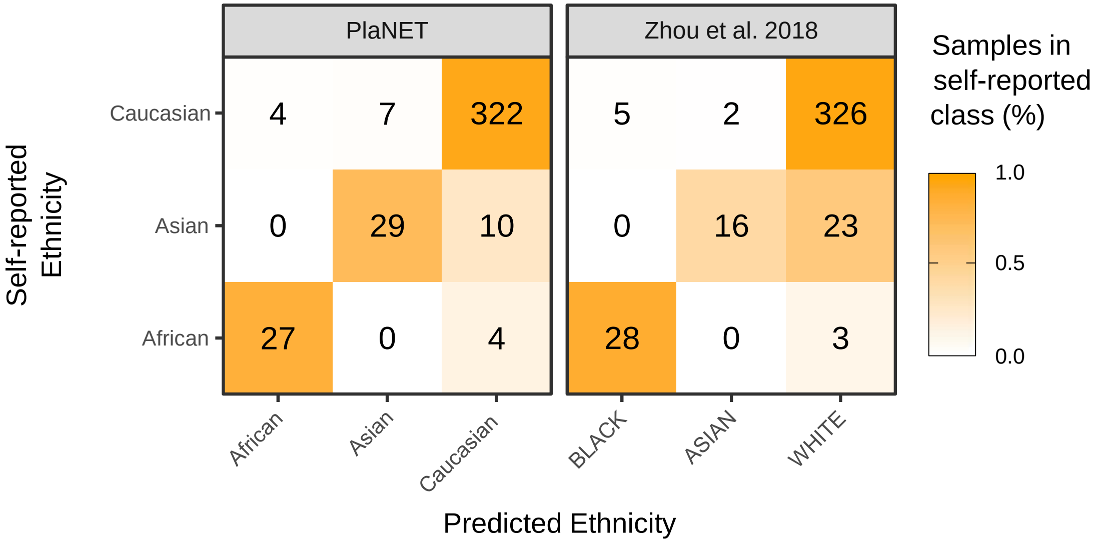

Figure S7. PlaNET vs Zhou et al. 2017 snp-based classifier. PlaNET's ethnicity classification performance was compared to Zhou et al. 2018 [76] SNP-based ethnicity classifier in cohorts C3, C4, and C5.

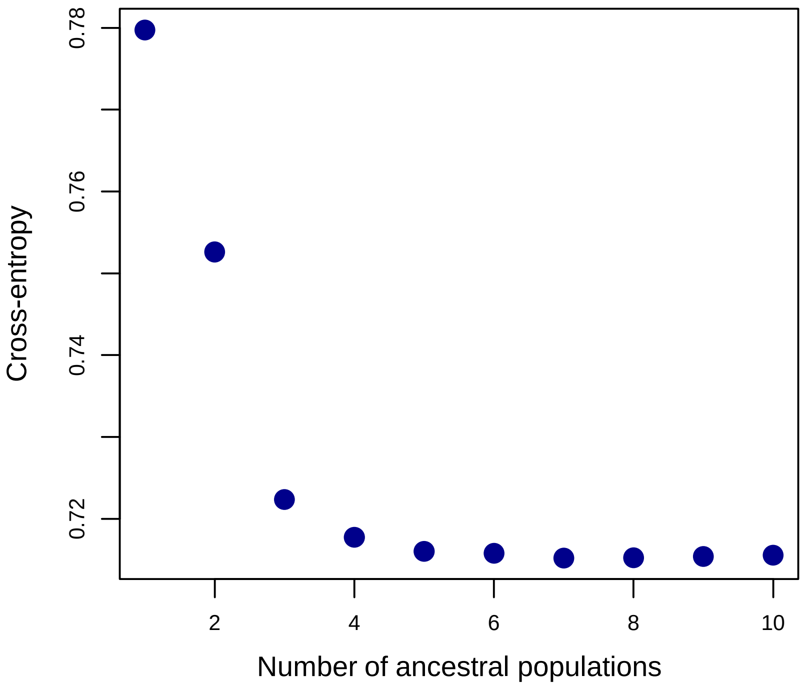

Figure S8. Estimating k number of ancestral populations using in genetic admixture inference program LEA. The cross-entropy criterion was used to determine the number of ancestral populations for estimating genetic ancestry coefficients. The number of ancestral populations was chosen at the point k = 3, when the cross-entropy criterion decreases signficantly less with each integer-increase in k.

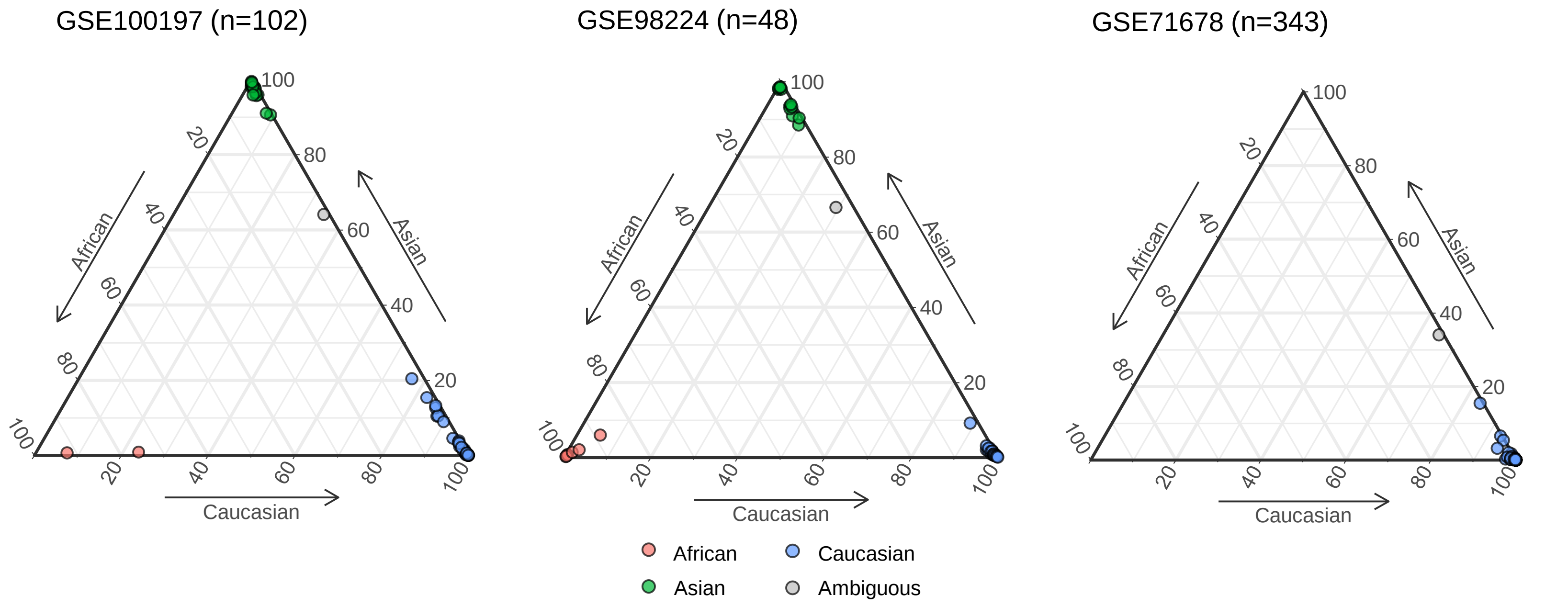

Figure S9. Application of PlaNET to placental EWAS. Samples from three independent cohorts are plotted along three axes by their probability of belonging to each ethnicity class and colored by their final ethnicity classification determined by PlaNET.

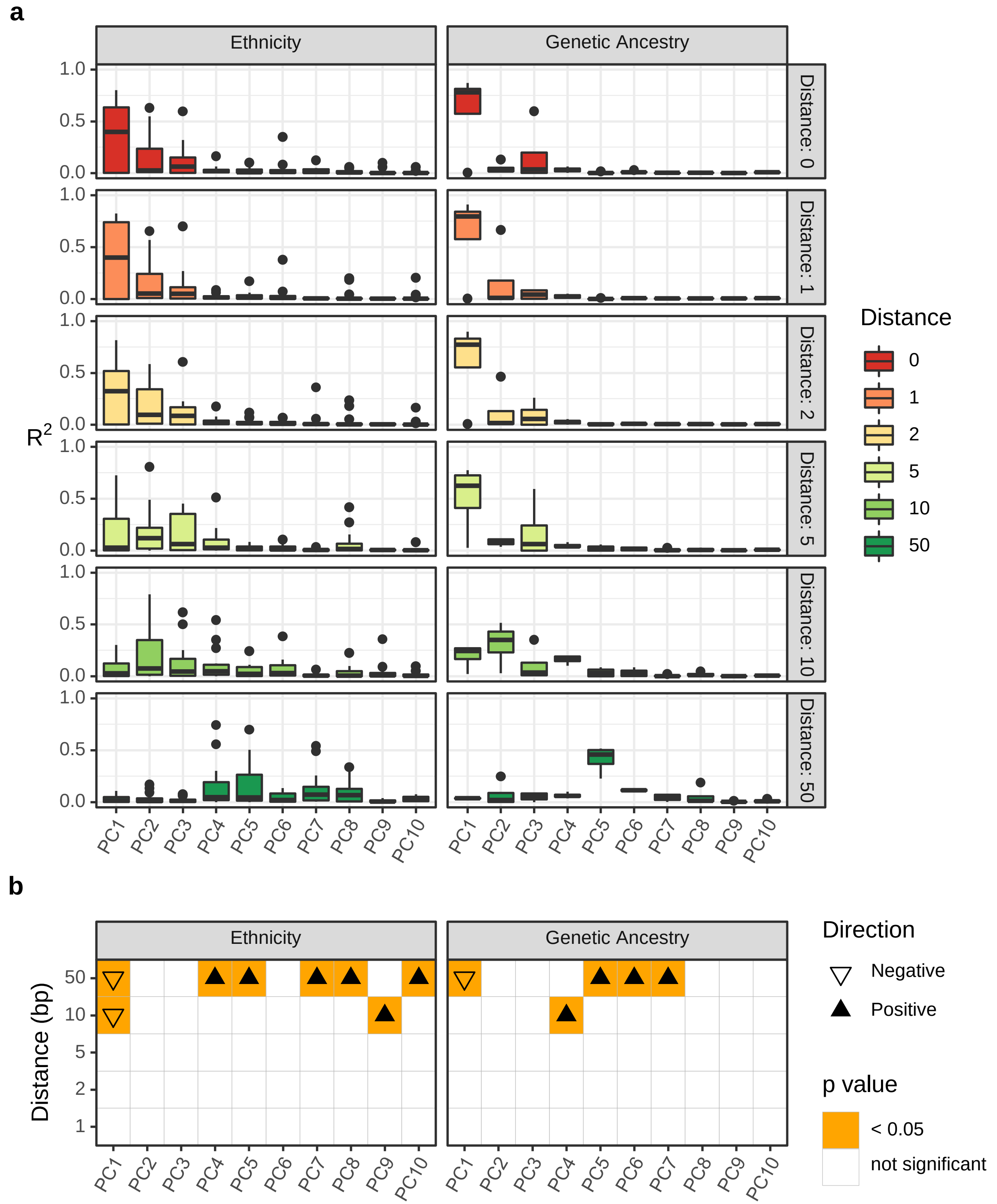

Figure S10. Evaluation of Barfield's alternative location-based filtering approaches. The signal associated with ethnicity and genetic ancestry was measured in relation to the distance of which a genetic variant lies to a CpG site (0, 1, 2, 5, 10, 50 bp). **a** Amount of variance explained in PCi (i = 1, 2, 3, ..., 10) by either ethnicity or genetic ancestry. **b** Whether there was difference in the amount of ethnicity or genetic ancestry -associated variation in PCi, depending on distance to a genetic variant. Direction of association is indicated, where the reference group is the 0 bp set.
